# Gingival phenotypes and their relation to age, gender and other risk factors

**DOI:** 10.1186/s12903-020-01073-y

**Published:** 2020-03-25

**Authors:** Wadhah Abdulnasser Alhajj

**Affiliations:** 1 Department of Periodontics, Faculty of Dentistry, Thamar University, Dhamar, Yemen; 2 Department of Dentistry, Faculty of Medical sciences, Civilization University, Sana’a, Yemen

**Keywords:** Phenotype, Gingiva, Keratinized, Papilla, Yemeni

## Abstract

**Background:**

Careful consideration and assessment of the type of phenotype has gained a fundamental importance in the treatment planning for any patient. We evaluated the prevalence of gingival phenotypes in a sample of Yemeni population and to explore its relationships to gender, age and other risk factors.

**Methods:**

This cross-sectional study was performed among 456 patients. All maxillary anterior teeth were included for all parameters and 1st molars were included for gingival thickness measurements. All patients included in this study were systemically healthy and presented no dental crowding. Four clinical parameters were systematically recorded: Gingival thickness (GT), Width of keratinized gingiva (WKG), Crown width/ crown length (CW/CL) ratio and Papilla height (PH). Scores obtained from different parameters measurements were recorded and analyzed using non-parametric tests where *P*-value < 0.05 was considered significant. One examiner performed all measurements.

**Results:**

The mean age was 29.9 ± 8.26 years. Of 456 recruited subjects, 83 (18.2%) subjects had thin, 69 (15.1%) had thick GT and 304 (66.7%) were placed in non-categorized (1.5–2 mm) GT. Square crown shape was found in 210 (44.1%) patients and 245 patients (55.9%) showed rectangular shape. Regarding WKG, 114 (25%) patients had width < 4 mm, 319 (70%) had width 4.1–8 mm and 23 (5%) patients had width >  8 mm. There was no significant difference between males and females for GT measurements. Regarding WKG, results showed that the prevalence of WKG 4.1–8 mm was more among females while males had more prevalence of ≤4 mm with significance difference. PH showed no significant differences between males and females. Regarding age, there was no significant differences between patients ≤25 years and >  25 years for all gingival parameters measurements. The relationship of smoking with different gingival parameters also showed no significant differences between smokers and non-smokers. Similarly, relationship of khat chewing with different gingival parameters showed no significant difference. Regarding inter-relationship between parameters, thin GT was associated with rectangular tooth form while square and quadrate forms are more associated with “1.5–2 mm” GT. WKG of ≤4 mm was associated with rectangular tooth form while WKG >  8 was more associated with square and quadrate forms with no significant difference. Results showed significant association between thin GT with 4.1–8 mm WKG.

**Conclusion:**

Yemeni population had more prevalence of “1.5–2 mm” GT, rectangular crown shape and WKG from 4.1–8 mm. Regarding interrelationship between gingival parameters, GT showed obvious relationship with WKG, CW/CL ratio and PH. WKG with CW/CL also showed significant relationship while no relationship was shown between other gingival phenotype parameters.

## Background

In 1969, Ochsenbein and Ross documented that two major kinds of gingiva morphology, named as: scalloped and thin or flat and thick gingiva [1]. Subsequently, the term “periodontal biotype” was advanced by Seibert and Lindhe to classify the gingiva into “thick-flat” and “thin-scalloped” biotypes [2]. Claffey and Shanley defined the thin tissue biotype as a GT of < 1.5 mm, and the thick tissue biotype was referred to as having a tissue thickness ± 2 mm (measurements of 1.6 to 1.9 mm were not accounted for) [3]. These gingival types could be recognized with a slightly scalloped gingival margin, short and wide teeth on the one hand and a thin, highly scalloped gingival margin with slender teeth on the other. In the World Workshop on the Classification of Periodontal and Peri-Implant Diseases and the term periodontal biotype was replaced by periodontal phenotype [4]. In clinical practice, the identification of the gingival phenotype is considered important because differences in gingival and osseous architecture have been shown to exhibit a significant impact on the outcome of restorative therapy [5]. Gingival phenotype back to a collection of four characteristics of the soft tissues and the teeth they surround that build up to a particular picture. These are: 1. GT (thick or thin): The tissue thickness in a bucco-palatal dimension. 2. The gingival width (width of keratinized tissue WKG: Which indicated the width of the keratinized tissue when measured from the gingival margin to the muco-gingival junction. 3. Papilla height (PH)/proportion: The gingival part that fits between teeth. 4. Crown width/height ratio CW/CL: Long, slender teeth tend to be associated with contact points away from the alveolar crest and long papillae that fill the embrasures [6].

Many methods were proposed to measure tissue thickness. These include direct measurements, probe transparency [7], ultrasonic devices [8], and, most recently, cone-beam computed tomography (CBCT) [8]. In the direct method, the tissue thickness was measured using a periodontal probe [7]. When the thickness was ±1.5 mm, it was categorized as a thick phenotype. When the thickness was < 1.5 mm, it was considered a thin tissue phenotype. The width of attached gingiva varies from tooth to tooth and also among individuals with mixed opinions regarding an “adequate” or “sufficient” dimension of the gingiva. Although the need for a so-called adequate amount of keratinized tissue for maintenance of periodontal health is questionable, the mucogingival junction serves as an important clinical landmark in periodontal evaluation. There are various methods of locating the mucogingival junction namely the functional method and the visual method with and without histochemical staining, which aid in the measurement of the width of attached gingiva [9].

Tissue phenotype are related to the response of the periodontal tissues to any physical, chemical, or bacterial insult, outcome of restorative, periodontal therapy, root coverage procedures, and overall esthetics of a dentition. Careful consideration and assessment of the type of phenotype has gained a fundamental importance in the treatment planning for any patient. Hence, it is important to gain knowledge about the prevalence of gingival phenotype in the general population and its relationship with other known clinical parameters [10]. The aims of this study was to evaluate the prevalence of gingival phenotypes in a sample of Yemeni population and assess its relationship to gender, age, smoking and khat chewing and other risk factors. This study also aimed to evaluate the interrelationship between the parameters of gingival phenotype.

## Methods

A Qualtrics ^XM^ sample size calculator was used to calculate the sample size based on the population size of the surveyed area. The power was calculated to be 95% using marginal error of 0.05. The total population size was considered as three millions which is the approximate enumeration of Sana’a city which is the site of our study. A total of 456 subjects were required for this study. Subjects were selected from a private clinic in Sana’a city. Each patient attended the clinic and coinciding the inclusion criteria was enrolled in the study. This research was approved by the ethics committee at Thamar University (Faculty of Dentistry) with a reference number (2019002). All maxillary anterior teeth were included for all parameters and 1st molars were included for GT measurements. All patients included in this study were systemically healthy and presented no dental crowding. The exclusion criteria were: (i) subjects with a mouth breathing habit (ii) subjects with crown restorations or fillings involving the incisal edge on anterior maxillary teeth, (iii) those with any removable device such as a removable partial denture, or removable orthodontic retainer, (iv) missing any of the six maxillary anterior teeth and having Millers Class III or Class IV recession (v) pregnant or lactating females and (vi) subjects taking medications with any known effect on the periodontal soft tissues [11, 12]. All subjects were provided with oral hygiene instructions and tooth polishing. This was preceded by calculus removal, if necessary. Witten informed consents were obtained from study participants.

### Clinical parameters

Four clinical parameters were systematically recorded by a single clinician 2 weeks following oral hygiene instructions and dental cleaning:-

**Fig. 1:**
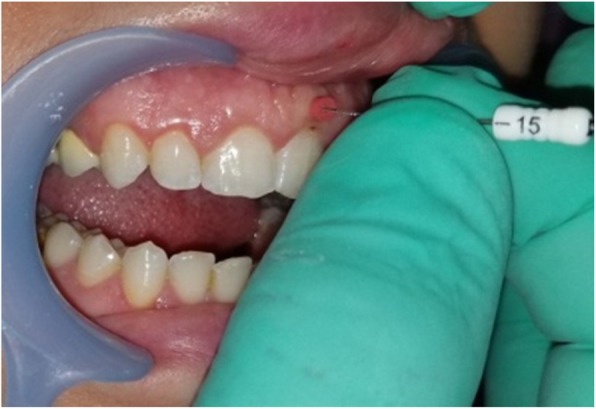
Measurement of GT with endodontic spreader No 15

**Fig. 2:**
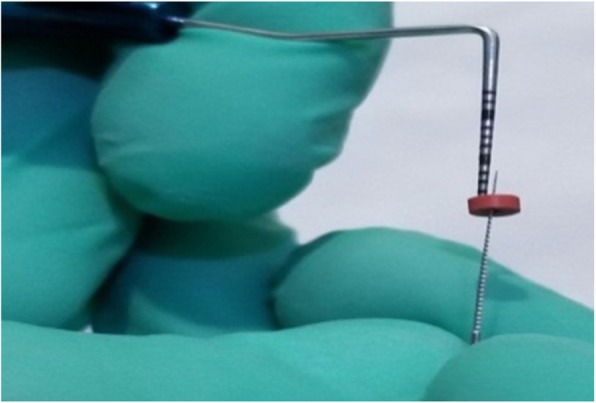
Measurement of penetration of gingiva with periodontal probe

**Fig. 3:**
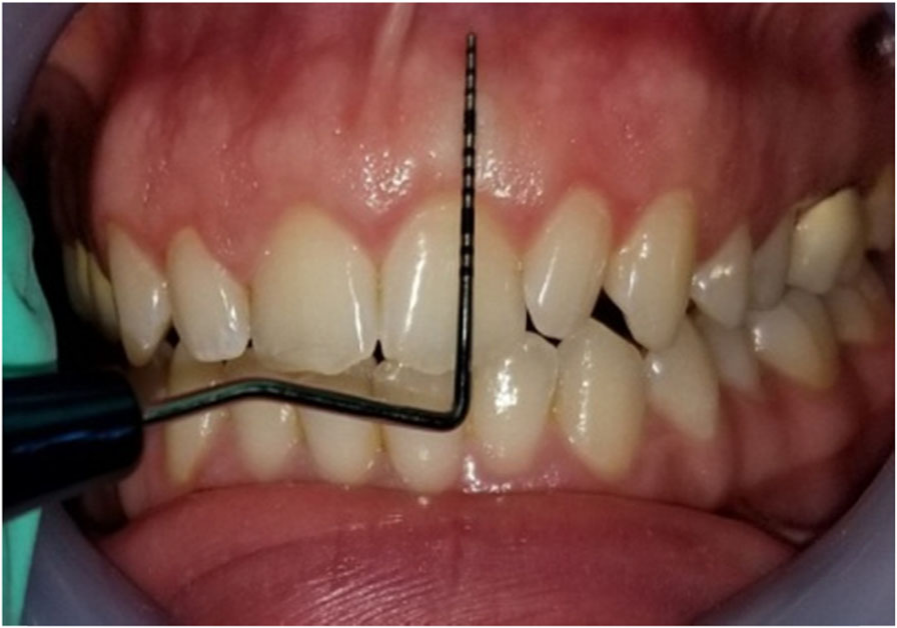
Measurement of WKG

**Fig. 4:**
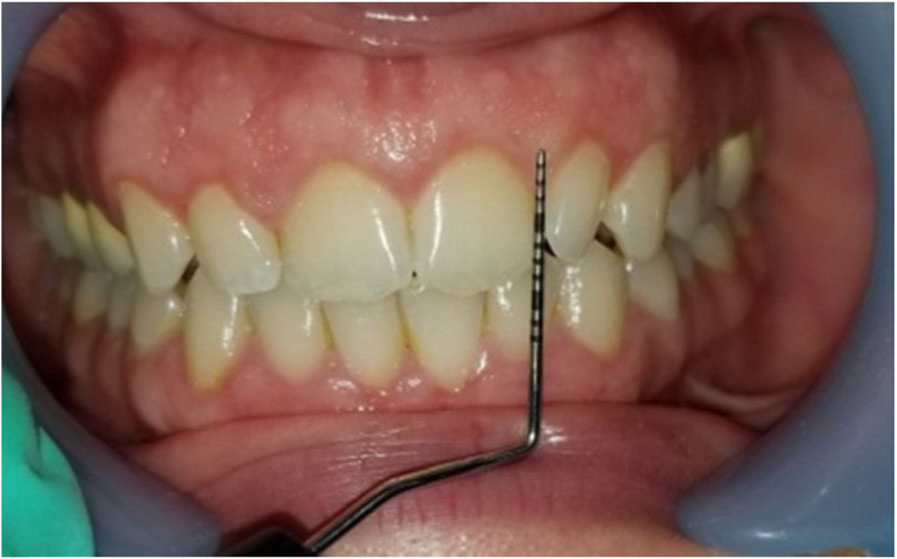
Measurement of PH

**Fig. 5:**
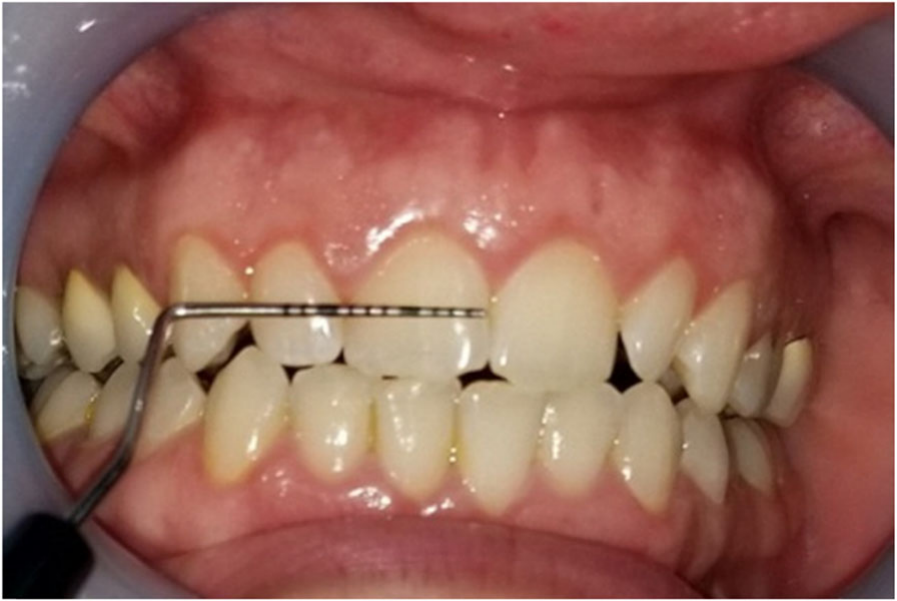
Measurement of crown width

- Gingival thickness (GT) was evaluated and categorized into thick or thin. This evaluation was based on direct measurements according *to Seibert and Lindhe* [2]. According to this classification, thickness between (1.5–2 mm) is not-categorized. The gingiva was anesthetized by topical-application of an anesthetic gel. An endodontic spreader size 15 with a rubber stop/caliper was inserted at a point at the center of the gingival margin and mucogingival junction in a perpendicular direction (Fig. 1) and this measurement was recorded with periodontal probe (Fig. 2) [13]. The thickness of the attached gingiva was recorded for upper right central incisor, upper left lateral incisor, upper right 1st molar, lower right 1st molar, upper left 1st molar and lower left 1st molar. The final readings for maxillary and mandibular GT were obtained by calculating the mean of all six measurements [14].
- Width of keratinized gingiva (WKG) was measured mid-facially to the nearest 0.5 mm according to *Fischer* et al. [15] with a periodontal probe (UNC 12) (Fig. 3). This parameter is defined as the distance from the free gingival margin to the mucogingival junction. So, we measured the keratinized gingival width from the gingival margin in the most apical point of the margin to mucogingival margin.
- Papilla height (PH) was assessed to the nearest 0.5 mm *Fischer* et al. [15] using the same periodontal probe at the mesial and the distal aspect of both central and lateral incisors (Fig. 4). This parameter is defined as the distance from the top of the papilla to a line connecting the mid-facial soft tissue margin of the two adjacent teeth [16]. The mean value was calculated for the five measured papillae.
- Crown width/crown length (CW/CL) ratio of the right central incisor was determined according to *Olsson & Lindhe* [16]. Assessments of width and length were recorded to the nearest 0.5 mm *Fischer* et al. [15] using a periodontal probe (Fig. 5). The crown length was measured between the incisal edge of the crown and the free gingival margin, or if discernible, the cemento-enamel junction. Crown width, i.e. the distance between the proximal tooth surfaces, was recorded at the border between the middle and the cervical portion.

As a ratio of 80:100 seems to be ideal, a CW/CL above 80% may be regarded as broad square and below as narrow rectangular [17]. In our study, (8\8–9\9–7\8–8\9 ratios) were considered as square or quadrate and (8\10–7\9–7.5 \9–8 \11) ratios were considered as rectangular. Scores obtained from measures of different parameters were recorded and averaged errors were minimized by allowing only one examiner to perform all the measurements.

### Statistical analysis

Descriptive statistics were performed in terms of means, frequencies, and percentages for study variables. Non-parametric Chi-Squared test was used for the association between the study variables. Contingency Chi-Squared test was used for variables with more than 2 categories. *P*-value less than 0.05 was considered significant for all tests. The Computer program software SPSS V22 for Windows was used for data analysis.

## Results

The mean age of the study population was 29.9 ± 8.26 years. Of the total 456 subjects, there were 215 (47.2%) males and 241 (52.8%) females; 83 (18.2%) subjects had thin GT, 69 (15.1%) had thick GT and 304 (66.7%) were present in non-categorized (1.5–2 mm) thickness. 210 (44.1%) patients demonstrated square or quadrate crown shape and 245 (55.9%) showed rectangular shape. Regarding WKG, 114 (25%) patients had width < 4 mm, 319 (70%) had width 4.1–8 mm and 23 (5%) patients had width >  8 mm. More details were shown in Table 1. All parameters showed no significant difference between the measured teeth. Table 2 shows the relationship of gender and age groups with different gingival parameters. There was no significant difference between males and females for GT and PH measurements while WKG results showed more prevalence of WKG 4.1–8 mm in females and males had prevalence of ≤4 mm with significance difference (*P* = 0.006). Regarding the relationship of age groups with different gingival parameters, there was no significant differences between patients ≤25 years and >  25 years for all gingival parameters. Relationship of smoking with different gingiva parameters also showed no significant difference between smokers and non-smokers. Duration of the smoking appeared as an important factor in GT measurements. Smoking with duration less than 5 years are more associated with thin GT while smoking more than 5 years showed more association with (1.5–2.0 mm) GT with significant difference. WKG and PH showed no significant difference between two groups (Table 3). The relationship of khat chewing with the different gingiva parameters also showed no significant differences between khat chewers and non-chewers in all parameters. Similarly, There were no significant differences between khat chewers < 5 years and ≥ 5 years for all gingival parameters (Table 4). The associations between the study variables are shown in Table 5. There was significant association between GT and WKG (*P* = 0.009), the results showed association between thin GT with 4.1–8 mm WKT. Also, significant association (*P* <  0.001) was shown between GT with CW/CL Ratio. In this regard, thin GT was associated with rectangular tooth form while square and quadrate forms were more associated with “1.5- 2 mm” GT. The association between GT with PH showed worthwhile results which revealed that thin GT was obviously associated with PH ≤ 3 mm and thick thickness was more associated with PH >  3 mm with significant difference (*P* = 0.011). Regarding the association between CW/CL with WKG, thickness of ≤4 mm was associated with rectangular tooth form while thickness >  8 mm was more associated with square and quadrate forms with significant difference (*P* = 0.005). Finally, PH failed to show an association with WKG although all cases of > 8 mm WKG had PH >  3 mm but other classes did not show any associations (*P* = 0.559). Similarly, no significant association was noticed between CW/CL with PH (*P* = 0.117).

**Table 1:** Characteristics of the study sample

Parameters	Frequency	%
Gender		
Male	215	47.2
Female	241	52.8
Age		
≤ 25 years	154	33.8
> 25 years	302	66.2
Smoking		
Yes	132	28.9
No	324	71.1
Duration		
< 5 years	61	46.2
≥ 5 years	71	53.8
Khat chewing		
Yes	221	48.5
No	235	51.5
Duration		
< 5 years	76	34.4
≥ 5 years	145	65.6
Gingival thickness		
< 1.5 mm	83	18.2
1.5–2 mm	304	66.7
> 2 mm	69	15.1
CW/CL Ratio		
Square/quadrate	210	44.1
Rectangular	246	55.9
Width of keratinized gingiva		
≤ 4 mm	114	25
4.1–8 mm	319	70
> 8 mm	23	5
Papilla height		
≤ 3 mm	250	54.8
> 3 mm	206	45.2

**Table 2:** Comparison between both genders and age groups in relation to GT, WKG, and PH

		Gender		*P*	Age		*P*
		Male	Female		≤ 25 years	> 25 years	
GT	< 1.5 mm	28 (13.0)	56 (23.2)	0.347	29 (18.8)	56 (18.5)	0.931
	1.5–2 mm	155 (72.2)	149 (61.8)		104 (67.5)	199 (65.9)	
	> 2 mm	32 (14.8)	36 (15.0)		21 (13.7)	47 (15.6)	
WKG	≤ 4 mm	81 (37.7)	34 (14.1)	0.006*	25 (16.2)	87 (28.8)	0.303
	4.1–8 mm	127 (59.1)	191 (79.3)		121 (78.6)	200 (66.2)	
	> 8 mm	7 (3.2)	16 (6.6)		8 (5.2)	15 (5.0)	
PH	≤ 3 mm	115 (53.5)	133 (55.2)	0.891	97 (63.0)	152 (50.3)	0.186
	> 3 mm	100 (46.5)	108 (44.8)		57 (37.0)	150 (49.7)

**Table 3:** Comparison between smokers and non-smokers and smoking duration in relation to GT, WKG, and PH

		Smoking		*P*	Smoking duration		*P*
		Yes	No		< 5 years	≥ 5 years	
GT	< 1.5 mm	33 (25.0)	48 (14.8)	0.112	27 (44.3)	5 (7.0)	0.007*
	1.5–2 mm	68 (51.5)	236 (72.8)		13 (21.3)	56 (78.9)	
	> 2 mm	31 (23.5)	40 (12.3)		21 (34.4)	10 (14.1)	
WKG	≤ 4 mm	23 (17.4)	93 (28.7)	0.246	4 (6.6)	19 (26.8)	0.354
	4.1–8 mm	95 (72.0)	219 (67.6)		47 (77.0)	47 (66.2)	
	> 8 mm	14 (10.6)	12 (3.7)		10 (16.4)	5 (7.0)	
PH	≤ 3 mm	82 (62.1)	168 (51.9)	0.345	26 (42.6)	56 (78.9)	0.093
	> 3 mm	50 (37.9)	156 (48.1)		35 (57.4)	15 (21.1)

**Table 4:** Comparison between Khat chewers and non-chewers and Khat chewing duration in relation to GT, WKG, and PH

		Khat chewing		*P*	khat chewing duration		*P*
		Yes	No		< 5 years	≥ 5 years	
GT	< 1.5 mm	34 (15.1)	46 (19.6)	0.803	13 (17.1)	21 (14.5)	0.719
	1.5–2 mm	149 (67.9)	156 (66.4)		55 (72.4)	95 (65.5)	
	> 2 mm	38 (17.0)	33 (14.0)		8 (10.5)	29 (20.0)	
WKG	≤ 4 mm	66 (30.2)	50 (21.2)	0.084	34 (44.7)	34 (23.5)	0.259
	4.1–8 mm	134 (60.4)	181 (77.0)		38 (50.0)	95 (65.5)	
	> 8 mm	21 (9.4)	4 (1.8)		4 (5.3)	16 (11.0)	
PH	≤ 3 mm	130 (58.5)	120 (51.0)	0.425	55 (72.4)	74 (51.0)	0.15
	> 3 mm	91 (41.5)	115 (49.0)		21 (27.6)	71 (49.0)

**Table 5:** Inter-associations between GT, WKG, and PH

		**GT**		
	**< 1.5 mm**	**1.5–2 mm**	**> 2 mm**	***P*-Value**
WKG				
≤ 4 mm	0 (0.0)	92 (30.3)	22 (31.9)	0.009*
4.1–8 mm	83 (100.0)	211 (69.4)	25 (36.2)	
> 8 mm	0 (0.0)	1 (0.3)	22 (31.9)	
CW/CL				
Square/Quadrate	0 (0.0)	187 (89.0)	23 (11.0)	< 0.001*
Rectangular	83 (33.7)	117 (47.6)	46 (18.7)	
PH				
≤ 3 mm	61 (73.5)	168 (55.3)	21 (30.4)	0.011*
> 3 mm	22 (26.5)	136 (44.7)	48 (71.6)	
		**WKG**		
	**≤ 4 mm**	**4.1–8 mm**	**> 8 mm**	
CW/CL				
Square/Quadrate	32 (15.2)	155 (73.8)	23 (11.0)	0.005*
Rectangular	82 (33.3)	164 (66.7)	0 (0.0)	
PH				
≤ 3 mm	58 (50.9)	192 (60.2)	0 (0.0)	0.599
> 3 mm	56 (49.1)	127 (39.8)	23 (100.0)	
		**CW/CL**		
	**Square/Quadrate**		**Rectangular**	
PH				
≤ 3 mm	129 (61.4)		121 (48.4)	0.117
> 3 mm	81 (38.3)		125 (51.6)	

## Discussion

The dimensions of gingiva and different parts of the masticatory mucosa demonstrate considerable site and subject variability. They have become the subject of considerable interest in restorative and periodontics from both an epidemiologic, as well as a therapeutic point of view [8]. In this study, the vast majority of cases had GT between (1.5–2.0 mm) with prevalence 66.7%. Thin and thick gingivae represents less prevalence in Yemeni population. Thin GT requires special considerations during esthetic, restorative, and periodontal therapy. These results are not correspondent to *Shah* et al. [10] and *Zawawi KH* et al. [18] who showed that thin GT was observed in 43.25 and 44.50% of the sample. These differences may be logical because they use different classifications. According to *Kydd* et al. [19], the average thickness of gingiva between lateral incisors and central incisors are slightly below 3 mm between premolars and molars slightly above 3 mm. These results are different from the results of present study with 1.9 mm for incisors and 1.8 mm for molars. *Shah* et al. [10] and *Egreja AM* et al. [13] recorded more convergent values with our study.

Regarding evaluation of the relationship of smoking and GT, our data revealed that non-smokers had more GT between (1.5–2.0 mm) while smokers had more thick GT (> 2 mm) but with no significant difference. Our results were correspondent to *Zawawi KH* et al [18] who have Only 31.4% of current smokers had thin GT. Our results showed that smoking with duration less than 5 years are more associated with thin GT while smoking more than 5 years showed more association with GT between (1.5–2.0 mm). To best to our knowledge, no documented studies discussed this topic. The relationship of GT with age was evaluated by *Agarwal V.* et al. [14] They observed that thickness of gingiva significantly decreased with age which contradicted the results of this study which showed that there is no difference in GT with age. *Agarwal V.* et al. [14] also studied the relationship of GT with gender and found that the thickness of gingiva significantly higher in females than males. Our results also disagreed with these results with males showed thicker GT than females. This contradiction may be due to ethinic differences where the mentioned study was done in India and this study was done in Yemen. *Muller’s*, *Wara-aswapati* et al. [20] and *Vandana and Savitha* [21] studies agreed with us and supported that women have thinner gingivae than men [22]. *Zawawi KH* et al [18] also stated that thin GT represents 64% in females and 25% in males. De rouck also agreed with us as he observed that thin gingiva was found mainly in female with slender teeth. Also, he found that thick gingiva was found mainly in males which is not correspondent to us with approximately equal distribution between males and females [5].

Evaluation of WKG showed that most of Yemeni population are categorized in 4.1–8 mm group which considered as sufficient keratinized gingiva. *Shah* et al. [10] documented that the mean WKG of central incisor, lateral incisor, and canine in Group I was 4.38 ± 1.18, 5.18 ± 1.25, 4.16 ± 1.16 mm respectively. This results were slightly less than our data with 5.0 ± 1.50, 5.9 ± 1.60 and 5.3 ± 1.36 for the same teeth but our data agreed with him in which WKG was the greatest for lateral incisor followed by central incisor and canine. When comparing this parameter between males and females, the results of the present study showed that females had more keratinized gingiva than males with significant difference. This may be in accordance to an explanation that stated that females were found to be twice as likely as males to have a gummy smile [22]. *De rouck* recorded that females had a narrow zone of keratinized tissue which contradicted our data [5]. *Shah* et al. [10] and *Olsson* et al. [16] also found a strong relationship between the WKG and GT which is correspondent to the results of present study. This finding further supports the notion that patients with a thin GT require a more careful treatment planning [10]. No relationship was found between WKG with age, where approximate keratinized gingiva where found in different age groups. This is conflicting with *Ainamo A.* et al. [23] who stated that there is a continuous growth through adult age of the basal bone with continuous widening of the band of attached gingiva in the male but not in the female cranium. This difference may be because in the mentioned study, the age was to 63 years old in contrary to 51 in this study. The crown form (CW/CL Ratio) in this study revealed that rectangular form of incisors are more prevalent than square or quadrant form in Yemeni population. Regarding the relationship of CW/CL Ratio with WKG, our results showed that the thickness of ≤4 mm is associated with rectangular tooth form while thickness >  8 os more associated with square and quadrate forms. Olsson et al. [16] were not able to find a statistically significant difference in GT between thick and thin gingivae based on crown shape defined by CW/CL ratio This is controversy to our results which demonstrated that rectangular shape is mostly associated with thin GT while square and quadrate shape are more associated with thick and (1.5–2.0 mm) GT.

*Ochsenbein and Ross* [1] believed that long-tapered teeth tend to have a thin-scalloped periodontium, whereas wide-square teeth have thick-flat periodontia. These results were correspondent to our data which showed that thin GT is associated with rectangular tooth form while square and quadrate forms are more associated with (1.5–2.0 mm) GT*. Olsson* et al. [7] contradict this and reported that no significant difference between narrow- and wide-crown forms with respect to the thickness of the free gingiva. *Fischer* stated that CW/CL is not a reliable parameter to assess the gingival phenotype, because according to the available data, there might be low and high scalloping, slender and broad teeth within one gingival phenotype [15]. In this study, the lengths of the interproximal dental papillae varied from 1 to 6 mm, with most of them at 3–4 mm which in agreement with *Chang* [24, 25] and *Chen MC* et al. [26] and showed a negative relationship between age and papilla height. Those results was correspondent to this study, which revealed that age did not significantly influence the papilla height. They also showed that sex did not significantly influence the presence of the interproximal dental papilla or its length as our results did. Kan et al. [27] observed a significant higher interproximal tissue height in the thick GT group compared with the thin GT group. These results were convergent with our data which revealed that thin GT is obviously associated with PH ≤ 3 mm and thick GT is more associated with PH >  3 mm with significant difference. Olsson et al. [16] also found a relationship between PH and GT. *Chen* et al. [26] found that the length of the interproximal dental papilla was significantly related to the width of the keratinized gingiva. These results were in contradiction to our results which showed no obvious relationship between these parameters. Recently, *Chow* et al. discussed the appearance of gingival papillae in relation to crown shape and GT. They found that GT was positively correlated with interproximal tissue height and hence with papillae appearance [28]. We agreed with them as thin GT was obviously associated with PH ≤ 3 mm and thick GT was more associated with PH >  3 mm with significant difference.

## Conclusion

It is concluded that Yemeni population has more prevalence of (1.5–2.0 mm) GT, rectangular crown shape and WKG from 4.1–8 mm. Regarding interrelationship between gingival parameters, GT showed strong relationship with WKG, (CW/CL) ratio and (PH). WKG with CW/CL Ratio also showed strong relationship while no relationship was shown between other gingival phenotype parameters. Studies with a heterogeneous population are needed to confirm the results presented in our study. Future research are needed to develop a more precise expanded and flexible classification system to classify and analyze gingival phenotypes parameters.

## Data Availability

The datasets supporting the findings of this article are available from the corresponding author.

## Abbreviations

GT: Gingival thickness
WKG: Width of keratinized gingiva
CW/CL: Crown width/ crown length ratio
PH: Papilla height
